# The Role of *Iex-1* in the Pathogenesis of Venous Neointimal Hyperplasia Associated with Hemodialysis Arteriovenous Fistula

**DOI:** 10.1371/journal.pone.0102542

**Published:** 2014-07-18

**Authors:** Akshaar Brahmbhatt, Evelyn NievesTorres, Binxia Yang, William D. Edwards, Prabir Roy Chaudhury, Min Kyun Lee, Hyunjoon Kong, Debabrata Mukhopadhyay, Rajiv Kumar, Sanjay Misra

**Affiliations:** 1 Vascular and Interventional Radiology Translational Laboratory, Department of Radiology, University of Cincinnati, Cincinnati, Ohio, United States of America; 2 Department of Lab Medicine and Pathology, University of Cincinnati, Cincinnati, Ohio, United States of America; 3 Division of Nephrology and Hypertension, Department of Medicine, University of Cincinnati, Cincinnati, Ohio, United States of America; 4 Department of Chemical & Biomolecular Engineering, University of Illinois at Urbana-Champaign, Champaign, Illinois, United States of America; 5 Department of Biochemistry and Molecular Biology, Department of Medicine, Mayo Clinic, Rochester, Minnesota, United States of America; 6 Division of Nephrology and Hypertension, Department of Medicine, Mayo Clinic, Rochester, Minnesota, United States of America; University of Louisville, United States of America

## Abstract

Arteriovenous fistulas (AVFs) used for hemodialysis fail because of venous neointimal hyperplasia (VNH). There are 1,500,000 patients that have end stage renal disease worldwide and the majority requires hemodialysis. In the present study, the role of the intermediate early response gene X-1 (*IEX-1*), also known as *IER-3* in the pathogenesis of VNH was evaluated. In human samples removed from failed AVF, there was a significant increase in *IEX-1* expression localized to the adventitia. In *Iex-1* ^−/−^ mice and wild type (WT) controls, chronic kidney disease was induced and an AVF placed 28 days later by connecting the carotid artery to jugular vein. The outflow vein was removed three days following the creation of the AVF and gene expression analysis demonstrated a significant decrease in vascular endothelial growth factor-A (*Vegf*-*A*) and monocyte chemoattractant protein-1 (*Mcp-*1) gene expression in *Iex-1* ^−/−^ mice when compared to WT mice (P<0.05). At 28 days after AVF placement, histomorphometric and immune-histochemical analyses of the outflow vein demonstrated a significant decrease in neointimal hyperplasia with an increase in average lumen vessel area associated with a decrease in fibroblast, myofibroblast, and Ly6C staining. There was a decrease in proliferation (Ki-67) and an increase in the TUNEL staining in *Iex-1* KO mice compared to WT. In addition, there was a decrease in *Vegf*-*A*, *Mcp-*1, and matrix metalloproteiniase-9 (*Mmp-9*) staining. *Iex-1* expression was reduced *in vivo* and *in vitro* using nanoparticles coated with calcitriol, an inhibitor of *Iex-1* that demonstrated that *Iex-1* reduction results in decrease in *Vegf-A*. In aggregate, these results indicate that the absence of *IEX-1* gene results in reduced VNH accompanied with a decrease in proliferation, reduced fibroblast, myofibroblast, and Ly6C staining accompanied with increased apoptosis mediated through a reduction in *Vegf-A*/*Mcp-1* axis and *Mmp-9*. Adventitial delivery of nanoparticles coated with calcitriol reduced *Iex-1* and VNH.

## Introduction

There are more than 1, 500,000 patients worldwide with end stage renal disease (ESRD) [1]. These patients require hemodialysis for removal of uremic toxins and control of volume. In order for effective hemodialysis to be performed, an optimally functioning hemodialysis vascular access is needed. The arteriovenous fistula (AVF) has become the preferred vascular access for chronic hemodialysis because it has lower infection and thrombosis rates with increased patency when compared to polytetrafluoroethylene (PTFE) grafts [2], [3]. AVF stenosis occurs at the outflow vein due to venous neointimal hyperplasia (VNH) with the one-year patency of AVFs being approximately 60% [4]. The stenosis requires frequent radiological and surgical intervention to prolong AVF function resulting in increased economic and health burden [3], [5].

There are many factors that contribute to VNH including changes in shear stress, oxidative stress, hypoxic injury, and inflammation [6]–[13]. These factors can mediate changes in matrix metalloproteinases (MMPs), vascular endothelial growth factor-A (VEGF-A), nitric oxide, platelet-derived growth factors (PDGF), fibroblast specific proteins, hypoxia inducible factor-1 alpha (HIF-1α), and monocyte chemoattractant protein-1 (MCP-1) that contribute to the pathogenesis of VNH [2], [6], [11], [13]–[16]. The changes in these mediators can lead to major transmural changes in the vessel. For example, MMPs can disrupt the vessel architecture and alter remodeling of the wall. Coupled with angiogenic stimuli acting through HIF-1α and VEGF-A such mediators lead to the differentiation of adventitial fibroblasts to myofibroblasts with subsequent proliferation, migration, and stenosis formation [15], [17]. The cascade of events is enhanced by the underlying uremia and inflammation of ESRD [8]–[10]. Our knowledge in this area is still incomplete as there are many other genes that contribute to the pathogenesis of VNH. Exploration of these genes could lead to new therapeutic targets.

The immediate early response gene X-1, *Iex-1*, also known as *Ier-3*, is a response gene that has been shown to modulate cell growth and differentiation in a variety of cells [18]–[20]. In cardiomyocytes, *Iex-1* has been shown to be anti-hypertrophic and lead to apoptosis [21]. In other studies, overexpression of *Iex-1* has been shown to cause proliferation mediated via a NF-κβ pathway in response to shear stress [22]. Finally, it has also been shown to lead to cellular proliferation in vascular smooth muscle cells [21], [23]. In AVFs, cellular proliferation, migration, and changes in apoptosis are associated with VNH. Taken collectively, we hypothesized that there is increased *Iex-1* expression in venous stenosis associated with AVF failure and that reducing *Iex-1* expression would result in a reduction in VNH.

In the present study, we investigated the role of *Iex-1* in AVF failure by examining its expression in failed human specimens removed from patients with AVF. Next, in *Iex-1* ^−/−^ and wild type (WT) mice we created a chronic kidney disease and placed an AVF and determined the role of *Iex-1* in VNH using histomorphometric analysis. We then investigated the expression of several proteins that have been implicated in venous stenosis formation including *Vegf-A*, *Mcp-1*, and *Mmp-9* in AVFs. *In vitro* experiments were performed using hypoxic fibroblasts to demonstrate that expression levels of *Iex-1* and *Vegf-A* increase in hypoxic injury. Finally, we demonstrate that calcitriol, known to reduce *Iex-1*, coated nanoparticles composed of polylactic-co-glycolic acid (nano-PLGA) can be used to reduce *Iex-1* expression in both *in vitro* and *in vivo* experimental models.

## Concise Materials and Methods

### Surgical resection of AVF tissue

Mayo Clinic and University of Cincinnati Institutional Review Board approval was obtained before obtaining tissue. Tissue, which was resected during surgical revision or thrombectomy, as obtained in paraffin embedded blocks. Written consent was received from the patients. The patient data was analyzed anonymously.

### Experimental animals

Approval from the Mayo Clinic Rochester Institutional Animal Care and Use committee was obtained before any procedures were performed. The maintenance and housing of the animals was performed in accordance with the Public Health Service Policy on Humane Care and Use of Laboratory animal [24]. Animals were kept at 12/12 hr light/dark cycles, 22°C, and 41% relative humidity with access to food and water ad libitum. Anesthesia was induced prior to all procedures using an intraperitoneal injection of ketamine hydrochloride (100 mg/kg) and xylazine (10 mg/kg) and maintained using ketamine hydrochloride (40 mg/kg) and xylazine (15 mg/kg). We induced chronic kidney disease by removing the right kidney followed by ligation of the blood supply to the upper pole of the left kidney as described elsewhere [14]. Four weeks after nephrectomy, an AVF was created between the carotid artery and the ipsilateral jugular vein. All procedures were performed under dissecting microscope (Zeiss Operating Microscope OPMI 6-SDFC, Oberkochen, Germany) [15], [25], [26]. *Iex-1* knockout and wild type animals were euthanized at days 3 and 28 after the creation of the AVF. The outflow vein was removed and used for qRT-PCR at day 3 and histomorphometric analyses at day 28. Serum BUN and creatinine were measured prior to nephrectomy, at the time of AVF placement and at the time of sacrifice.

For testing the effects of nanoparticles composed of poly (lactic-*co*-glycolic) acid (PLGA) and coated with calcitriol, C57BL/6 mice were used and underwent nephrectomy followed by the placement of AVF twenty-eight days later as described previously. At the time of AVF creation, animals received either: hydrogel with PLGA, hydrogel with calcitriol+ nano PLGA or hydrogel alone. Animals were sacrificed at day 7 for qRT-PCR analysis and day 28 for histomorphometric analysis.

### Procedures to ensure animal comfort and anesthesia

Mayo Clinic ensures that all USDA regulations and the NIH guidelines for the care and use of laboratory animals are strictly enforced. All investigators are required to administer the appropriate analgesics to all animals during a procedure that would normally require pain medication in humans. The mice that undergo surgery for the creation of the AVF procedure were anesthetized by administering anesthesia as previously described per IACUC recommendations. All surgical procedures were conducted in a disinfected, uncluttered area, which promotes asepsis during surgery. The animals were maintained at a surgical plane of anesthesia throughout the procedure and the vital signs were monitored. The surgical incision was closed using appropriate techniques and materials. After surgery the animal was moved to a warm, dry area and monitored during recovery. Heat lamps or warming pads were used in maintaining or recovering body temperature.

### *IEX-1* null mutant mouse and genotyping

The mouse ortholog of the *Iex-1* gene is known as *Iex-1/gly96*. This knockout mouse was created using the *Iex-1/gly96* null mutant mouse [27]. These mice have been known to have hypertension and endothelial changes such as peliosis in the spleen [28]. Tail veins were removed from the mice at the time of weaning. The Qiagen DNeasy kit (Qiagen, Gaithersburg, MD) was used to process the tissue. DNA was then amplified using *Iex-1* primers and subsequently run out on an agarose gel to determine genotype. Male *Iex-1* ^−/−^ and WT mice were only used for the current study.

### Tissue harvesting

At the time of sacrifice, anesthesia was induced as described earlier. Mice were euthanized by CO^2^ asphyxiation. The fistula was dissected free and specimens were saved for qRT-PCR or histologic analysis as described elsewhere [15], [25].

### Calcitriol loaded poly (lactic-*co*-glycolic) acid (PLGA) nanoparticles and hydrogel

PLGA nanoparticles were encapsulated with calcitriol using the interfacial deposition method. Briefly, 100 mg of PLGA (Durect, Cupertino, CA.) and 0.1 mg of calcitriol (Tocris Bioscience, Bristol, UK) were dissolved in 10-mL of acetone. Then, the solution was added drop wise to deionized water. After particle formation, the organic solvent was evaporated and the PLGA nanoparticles were dried through lyophilization. The dried PLGA nanoparticles were dispersed in an aqueous solution at a concentration of 200-µM. For the PLGA particles alone an equivalent portion was made into a 200-µM solution. A 40% solution of pluronic F-127 (Sigma-Aldrich, St. Louis, MO) was prepared under sterile conditions at 4°C. Forty grams of pluronic F-127 were added to 100-mL of distilled water. This was placed on a stir plate and allowed to mix overnight at 4°C. This was then mixed with equal parts of a 200-µM PLGA or 200-µM of calcitriol+ PLGA solution. This resulted in two solutions of 20% hydrogel at 100-µM, which were kept on ice until the time of surgery. Each animal received of 5-µL of 100-µM calcitriol (208.32-ng) bound to nanoparticle in 20-wt% pluronic gel. At the time of surgery the solutions were warmed to room temperature before being applied to the adventitia of the outflow vein.

### Cell culture

To determine the expression of *Iex-1* and *Vegf-A in vitro*, NIH 3T3 cells (ATCC, Manassas, VA) were used and subjected to hypoxia and normoxia for different lengths of time as described previously [15], [25]. Cells were harvested for qRT-PCR analysis as previously described.

### RNA isolation

Cells or tissue were stored in a RNA stabilizing/cell lysis solution (Qiagen, Gaithersburg, MD) [15], [25]. Specimens were homogenized and total RNA was extracted and isolated via the miRNEasy Mini kit (Qiagen). Culture cells were trypsinized and stored in Qiazol lysis regent and processed using the RNEasy Mini kit.

### qRT-PCR analysis

Gene expression was quantified using qRT-PCR as previously described [15], [25]. Specific primers (Integrated DNA Technologies, Coralville, IA) used in this analysis are shown in table 1.

**Table 1:** Primers used.

Gene	Sequence
*18S*	5′-AGCTAGGAATAATGGAATAG-3′ (sense)
	5′-AATCAAGAACGAAAGTCGGAG-3′ (antisense)
*Iex-1*	5′-GCGCGTTTGAACACTTCTC-3′ (sense)
	5′-ATGGCGAACAGGAGAAAGAG-3′ (antisense)
*Mmp-9*	5′-GTTTTTGATGCTATTGCTGAGATCCA-3′ (sense)
	5′-CCCACATTTGACGTCCAGAGAAGAA-3′ (antisense)
*Mcp-1*	5′-GTCCCTGTCATGCTTCTGG-3′ (sense)
	5′-GCTCTCCAGCCTACTCATTG-3′ (anti sense
*Vegf-A*	5′-ATGAAGTGATCAAGTTCATGG-3′ (sense)
	5′-GGATCTTGGACAAACAAATGC-3′ (antisense)

### Tissue processing and immunohistochemistry

The outflow vein from each animal was embedded in paraffin lengthwise. Eighty to 120, 4-µm sections from each outflow vein from each animal are obtained and every 40-µm, 2–4 sections are stained with hematoxylin and eosin. In addition, Ki-67, α-SMA, Fsp-1, Mmp-9, Ly6C, Mcp-1, or Vegf-A staining was performed using EnVision (DAKO, Carpinteria, CA) method with a heat-induced antigen retrieval step for 30 minutes at 95-°C in sodium citrate solution. Four-µm sections orthogonal to the long axis were taken to show the lumen of the vessel as previously described by our group [14], [15]. The following antibodies were then used: mouse monoclonal antibody Ki-67 (DAKO, Carpinteria, CA, 1∶400), rabbit polyclonal antibody to mouse for rabbit polyclonal antibody to mouse for Vegf-A (Abcam, 1∶600), rabbit polyclonal antibody to mouse for Mmp-9 (Abcam, 1∶600), rabbit polyclonal antibody to mouse for Fsp-1 (Abcam, 1∶600), rabbit polyclonal antibody to mouse for α-SMA (Abcam, 1∶600), rabbit polyclonal antibody to mouse for Mcp-1 (Abcam, 1∶600), or rabbit polyclonal antibody to mouse for Ly6C (Abcam, Cambridge, MA; 1∶400). IgG antibody staining (1∶200) was performed to serve as a control.

### TUNEL

TUNEL (TdT-mediated dNTP nick end labeling assay) was performed on paraffin-embedded sections from the outflow vein of *Iex-1* KO or WT as specified by the manufacturer (DeadEnd Colorimetric tunnel assay system, G7360, Promega, Madison, WI). Negative controls were used when the recombinant terminal deoxynucleotidyl transferase enzyme was not used.

### Morphometric and image analysis

Sections that had been immunostained with hematoxylin and eosin were viewed using a Neo-Fluor x 20/0.50 objective lens on an Axioplan 2 Microscope (Zeiss, Oberkochen, Germany) [15], [25].

### Statistical analysis

Data are expressed as mean ± SEM. Multiple comparisons were performed with two-way ANOVA followed by Student *t*-test with post hoc Bonferroni's correction. Significant difference from control value was indicated by *P<0.05. JMP version 9 (SAS Institute Inc., Cary, N.C.) was used for statistical analyses.

## Results

### IEX-1 expression is significantly increased in failed clinical samples of AVF

**Figure 1:**
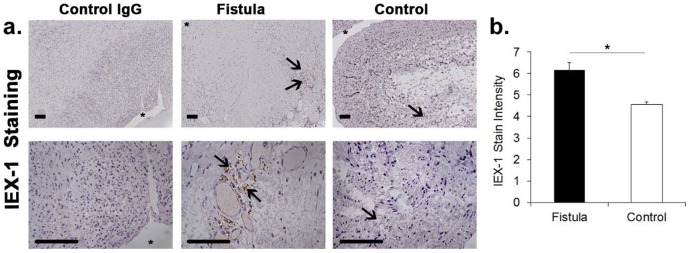
IEX-1 expression in malfunctioning AV fistulae when compared to controls removed from patients. Representative IEX-1 staining (**a**) is shown in a on outflow veins removed from patients with malfunctioning AVF when compared to control veins removed from patients undergoing placement of a AVF. IEX-1 expression was localized to the adventitia (arrow heads) of AVF when compared to controls. Brown staining cells are positive for IEX-1. Top row is 10X and bottom row is 40X. Scale bar is 100-µms. * denotes vessel lumen. Pooled data from the semiquantitative analysis for intensity of IEX-1 staining in the vessel wall of the outflow vein specimens removed from patients with AVF or controls is shown in **b.** This demonstrates a significant increase in the mean IEX-1 expression in the AVF when compared to controls (P<0.05). Each bar shows the mean ± SEM of 3 samples per group. Two-way Student t-test was performed. Significant difference from control value is indicated by * P<0.05.

The expression of *IEX-1* in malfunctioning hemodialysis vascular access remains unknown. The expression of *IEX-1* was analyzed using immunostaining in venous samples removed from patients with AVFs due to infection, thrombosis, or stenosis (n = 3, 2 M, average age  = 58.7±10.6 years old) and compared to control veins removed from patients (n = 3, 2 M, average age  = 60±2.6 years old) undergoing placement of AVF (**Fig. 1a**). There was a significant increase in the mean expression of *IEX-1* in AVF when compared to controls (**Fig. 1b**, average increase: 133%, 6±1.2 vs. 4.4±0.2, P<0.05, venous stenosis vs. control veins). Expression of *IEX-1* was localized to the adventitia of the vein (arrow).

### Surgical Outcomes

We used a strain of C57BL/6 mice that had a double allele knockout for the *Iex-1* gene as described elsewhere [28]. Only male mice with the following genotype were used: *Iex-1* ^−/−^ and *Iex-1* ^+/+^ (wild type, WT) mice. A total of 36 mice, liter and age matched, weighing 20–25 g had CKD induced by nephrectomy and a carotid artery to jugular AVF placed four weeks later as described previously by our group [15], [25]. Of the 36 mice, one mouse died after nephrectomy and two had a thickened artery that precluded the animals from having an AVF placed. Thirty-three mice [*Iex-1* ^−/−^ (n = 17) and wild type [*Iex-1* ^+/+^ (n = 16)] underwent AVF placement. Of these mice, 11 mice [*Iex-1* ^−/−^ (n = 6) and wild type *Iex-1* ^+/+^ (n = 5)] were sacrificed at day 3 for qRT-PCR analysis and twenty-two mice [*Iex-1* ^−/−^ (n = 11) and wild type *Iex-1* ^+/+^ (n = 11)] at day 28 for histomorphometric and immmuno-histochemical analysis.

We tested the efficacy of a known modulator of *Iex-1*, calcitriol, in preventing AVF stenosis by loading calcitriol onto nanoparticles composed of PLGA. The efficacy of calcitriol in reducing *Iex-1* expression using qRT-PCR was determined in C57BL/6 mice with established CKD and AVF by delivering the drug to the adventitia of the outflow vein at the time of AVF creation. We have previously delivered lentiviral tagged shRNA inhibitors to the adventitia with a similar approach [15], [29]. Twenty-two C57BL/6 male mice, 20–25 g, had CKD induced followed by a AVF placed to connect the carotid artery to the jugular vein twenty-eight days later. Animals were sacrificed at day 7 for qRT-PCR analysis (hydrogel alone, hydrogel with PLGA + Calcitriol, or hydrogel with PLGA alone) and day 28 for histomorphometric analysis (hydrogel alone, hydrogel with PLGA + Calcitriol).

### Serum BUN, creatinine, and blood pressure

There was no difference in the kidney function (BUN or creatinine) or blood pressure at baseline, after nephrectomy, or at AVF placement between the different groups of animals used.

### *Iex-1* ^−/−^ mice have decreased gene expression of *Vegf-A* and *Mcp-1* at 3 days after AVF placement

**Figure 2:**
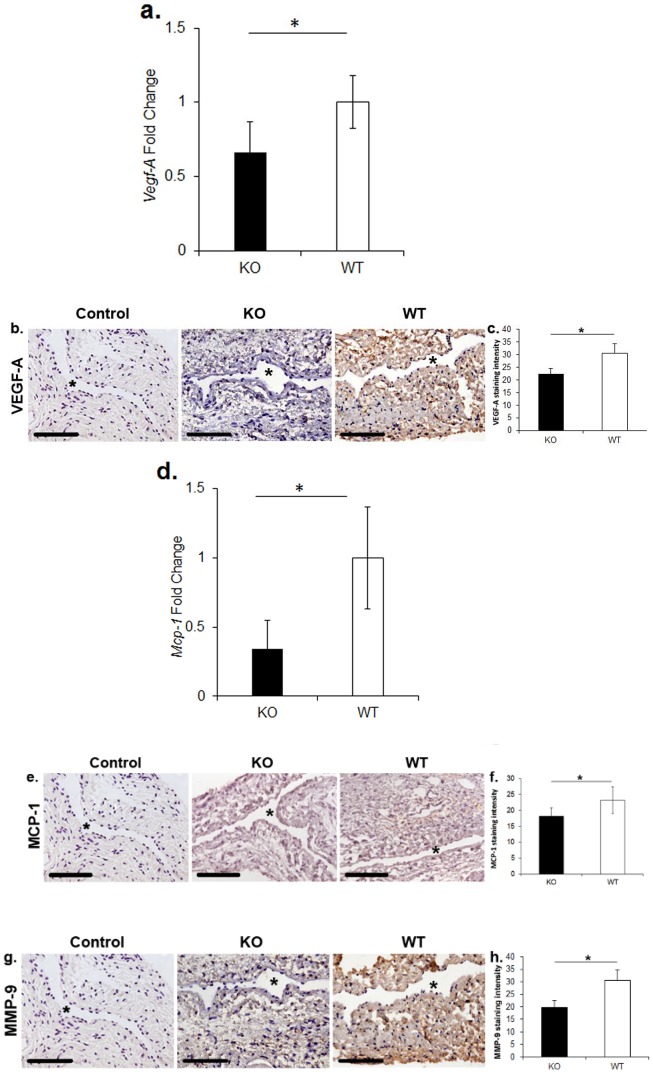
Gene expression of *Vegf-A* and *Mcp-1* by qRT-PCR at the outflow vein of AVF three days after fistula placement in *Iex-1* KO and WT mice. Immunohistochemical staining for VEGF-A, MCP-1, and MMP-9 at twenty-eight days after AVF placement in Iex-1 KO and WT mice. **a.** Pooled data for *Vegf-A* expression by qRT-PCR in outflow veins of AVF three days after fistula placement. There is a significant decrease in the average gene expression of *Vegf-A* in the *Iex-1* KO animals when compared to WT controls (P<0.05). **b.** Representative staining for VEGF-A on sections removed from the outflow vein of *Iex-1* KO (**second column**) and WT controls (**third column**) is shown. IgG antibody staining was performed to serve as negative control in the **first column**. Brown staining cells are positive for VEGF-A. **c.** Pooled data from the semiquantitative analysis for intensity of VEGF-A staining in the vessel wall of the outflow vein specimens removed from *Iex-1* KO and WT mice. There is a significant decrease in the mean VEGF-A staining in the *Iex-1* KO animals when compared to WT controls (P<0.05). **d.** Pooled data for the average gene expression of *Mcp-1* by qRT-PCR in outflow veins of AVF three days after fistula placement. There is a significant decrease in the expression of MCP-1 in the *Iex-1* KO animals when compared to WT controls (P<0.05). **e.** Representative staining for MCP-1 on sections on the outflow vein removed from *Iex-1* KO (**second column**) and WT controls (**third column**) are shown. IgG antibody staining was performed to serve as negative control in the **first column**. Brown staining cells are positive for MCP-1. **f.** Pooled data from the semiquantitative analysis for intensity of MCP-1 staining in the vessel wall of the outflow vein specimens removed from *Iex-1* KO and WT mice. There is a significant decrease in the mean MCP-1 staining in the *Iex-1* KO animals when compared to WT controls (P<0.05). **g.** Representative staining for MMP-9 sections from the outflow vein removed from *Iex-1* KO (**second column**) and WT controls (**third column**) are shown. IgG antibody staining was performed to serve as negative control in the **first column**. Brown staining cells are positive for MMP-9. **h.** Pooled data from the semiquantitative analysis for intensity of MMP-9 staining in the vessel wall of the outflow vein specimens removed from *Iex-1* KO and WT mice. There is a significant decrease in the mean MMP-9 staining in the *Iex-1* KO animals when compared to WT controls (P<0.05). For all representative sections * denotes vessel lumen. 40X magnification, scale bar is 100-µms.

Previous data from our laboratory has demonstrated that there is increased gene expression of *Vegf-A* at the venous stenosis [15], [25]. We have reduced *Vegf-A* expression and observed a decrease in *Mmp-9* in *Vegf-A* transduced vessels and *Mmp-9* is increased in experimental models of AVF failure [6], [14], [30], [31]. There have been studies that have demonstrated an interaction between *Vegf-A* and *Mcp-1* and moreover *Mcp-1* has been shown to contribute to VNH in experimental animal models [16]. We determined the gene expression of *Vegf-A* and *Mcp-1* on venous stenosis removed *Iex-1* ^−/−^ and WT controls at 3 days after AVF placement and protein expression using immunostaining at 28 days. As expected, the average gene expression of *Iex-1* was significantly reduced in *Iex-1* ^−/−^ mice when compared to WT controls (average decrease: 96%, 0.04±0.02 vs. 1±0.22, *Iex-1* ^−/−^ vs. WT, respectively, P<0.05) (data not shown). The average gene expression of *Vegf-A* in outflow vein removed from *Iex-1* ^−/−^ mice when compared to WT and found it was significantly decreased in the *Iex-1* ^−/−^ mice when compared to WT (average decrease: 34%, 0.66±0.21 vs. 1.0±0.17, *Iex-1* ^−/−^ vs. WT, respectively, P<0.05, **Fig. 2a**). Immunostaining for VEGF-A (**Fig. 2b**) was assessed at day 28 which demonstrated that the average VEGF-A staining was also significantly reduced in the *Iex-1* ^−/−^ mice when compared to WT controls (average decrease: 27%, 22.3±2.3 vs. 30.5±3.9, respectively, P<0.05, **Fig. 2c**).

Next, we determined the levels of *Mcp-1*. The gene expression of Mcp-1 was significantly reduced in *Iex-1* ^−/−^ mice when compared to WT controls (average decrease: 66%, 0.34±0.2 vs. 1±0.37, *Iex-1* ^−/−^ vs. WT, respectively, P<0.05, **Fig. 2d**). The average MCP-1 staining (**Fig. 2e**) was significantly reduced in the *Iex-1* ^−/−^ mice when compared to WT controls (average decrease: 27%, 22.3±2.3 vs. 30.5±3.9, respectively, P<0.05, **Fig. 2f**).

There was no difference in the gene expression of *Mmp-9* at day 3 in the *Iex-1* ^−/−^ mice when compared to WT controls. However, there was a significant decrease in the average MMP-9 staining (**Fig. 2g**) in venous stenosis removed from *Iex-1* ^−/−^ mice when compared to WT controls (average decrease: 35%, 19.8±2.7 vs. 30.6±4.5, *Iex-1* KO vs. WT, respectively<0.05, **Fig. 2h**).

### Outflow veins removed from *Iex-1* ^−/−^ mice have decreased neointima area, decreased cell density, and increased lumen vessel area

**Figure 3:**
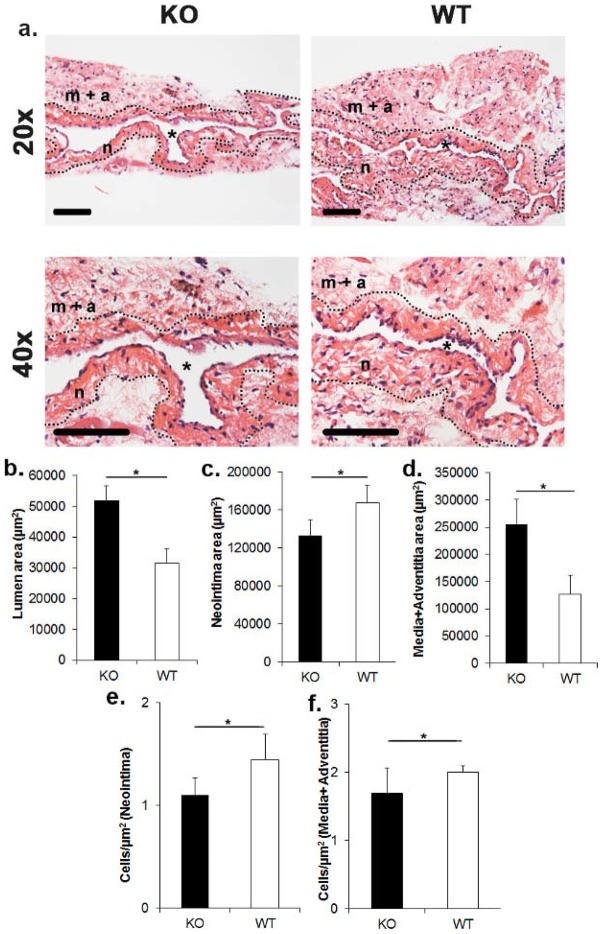
Hematoxylin and eosin staining with histomorphometric analysis of outflow vein removed from Iex-1^−/−^ and WT animals at day 28 after fistula placement. Representative hematoxylin and eosin staining of the outflow vein removed from *Iex-1* KO and WT mice from 28 days after fistula placement Is shown in **a.** The neointima (**n**) is identified from the media and adventitia by the dotted line. m+a is the media and adventitia. * denotes vessel lumen. Top row is 20X and bottom row is 40X. Scale bar is 100-µms. Pooled data from the mean lumen vessel area from *Iex-1* KO and WT animals are shown in **b.** There is a significant increase in the mean lumen vessel area in the *Iex-1* KO animals when compared to WT controls (P<0.05). Pooled data from the mean neointima area from *Iex-1* KO and WT animals are shown in **c.** There is a significant decrease in the mean area of the neointima in the *Iex-1* KO animals when compared to WT controls (P<0.05). Pooled data from the mean area of the media and adventitia from *Iex-1* KO and WT animals are shown in **d.** There is a significant increase in the mean area of the media and adventitia in the *Iex-1* KO animals when compared to WT controls (P<0.05). Pooled data from the mean cell density in the neointima from *Iex-1* KO and WT animals are shown in **e.** There is a significant decrease in the mean cell density of the neointima in the *Iex-1* KO animals when compared to WT controls (P<0.05). Pooled data from the mean cell density in the media and adventitia from *Iex-1* KO and WT animals are shown in **f.** There is a significant increase in the mean cell density of the media and adventitia in the *Iex-1* KO animals when compared to WT controls (P<0.05). Student *t*-test with post hoc Bonferroni's correction was performed. Significant difference from control value is indicated by * P<0.05. Each bar shows mean ± SEM of 4-5 animals per group.

Histomorphometric analysis of outflow veins removed from *Iex-1* ^−/−^ and WT mice were evaluated twenty-eight days after fistula placement. Using hematoxylin and eosin staining, we could differentiate between the neointima (**n**) and media and adventitia (**m+a**, **Fig. 3a**). The mean lumen vessel area was significantly increased in the *Iex-1* ^−/−^ mice when compared to WT controls (average increase: 144%, respectively, P<0.05, **Fig. 3b**) with a significant decrease in the average area of the neointima (average decrease: 20%, P<0.05, **Fig. 3c**). In addition, there was a significant increase in the average area of the media and adventitia (average increase: 201%, *Iex-1* ^−/−^ vs. WT, respectively, P<0.05, **Fig. 3d**). The average cell density was significantly decreased in the neointima of *Iex-1* ^−/−^ mice when compared to WT controls (average reduction: 25%, P<0.05, **Fig. 3e**) while in the media and adventitia, there was a 155% increase in the *Iex-1* ^−/−^ mice when compared to WT controls (P<0.05, **Fig. 3f**).

### There is a decrease in the expression of fibroblast, myofibroblast, and Ly6C staining in venous stenosis of *Iex-1* ^−/−^ mice when compared to WT controls at 28 days after AVF placement

**Figure 4:**
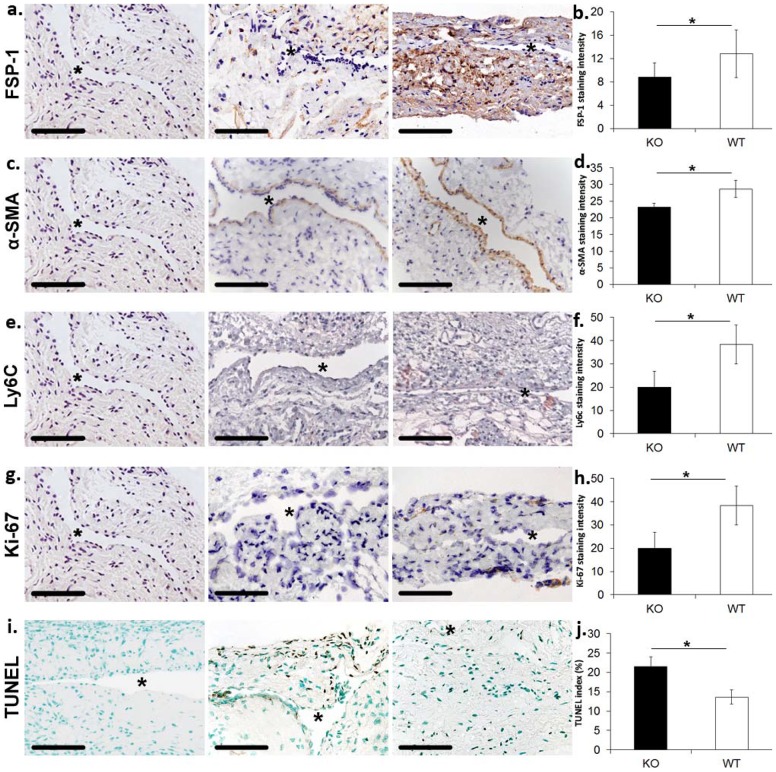
There is a significant decrease in fibroblast, α-SMA, and Ly6C staining accompanied with a decrease in proliferation, and increase in cell death in the outflow vein removed *Iex-1* KO mice when compared to WT controls at day 28 after fistula. **a.** Representative staining for Fsp-1 from the outflow vein removed from *Iex-1* KO (**second column**) and WT controls (**third column**) are shown. IgG antibody staining was performed to serve as negative control in the **first column**. Brown staining cells are positive for Fsp-1. All images are 40X. Scale bar is 100-µms. * indicates lumen. **b.** Pooled data from the semiquantitative analysis for intensity of Fsp-1 staining in the vessel wall of the outflow vein specimens removed from *Iex-1* KO and WT mice. There is a significant decrease in the mean Fsp-1 staining in the *Iex-1* KO animals when compared to WT controls (P<0.05). **c.** Representative staining for α-SMA from the outflow vein removed from *Iex-1* KO (**second column**) and WT controls (**third column**). IgG antibody staining was performed to serve as negative control in the **first column**. Brown staining cells are positive for α-SMA. **d.** Pooled data from the semiquantitative analysis for intensity of α-SMA staining in the vessel wall of the outflow vein specimens removed from *Iex-1* KO and WT mice. There is a significant decrease in the mean α-SMA staining in the *Iex-1* KO animals when compared to WT controls (P<0.05). **e.** Representative staining for Ly6C from the outflow vein removed from *Iex-1* KO (**second column**) and WT controls (**third column**). IgG antibody staining was performed to serve as negative control in the **first column**. Brown staining cells are positive for Ly6C. **f.** Pooled data from the semiquantitative analysis for intensity of Ly6C staining in the vessel wall of the outflow vein specimens removed from *Iex-1* KO and WT mice. There is a significant decrease in the mean Ly6C staining in the *Iex-1* KO animals when compared to WT controls (P<0.05). **g.** Representative staining for Ki-67 from the outflow vein removed from *Iex-1* KO (**second column**) and WT controls (**third column**). IgG antibody staining was performed to serve as negative control in the **first column**. Nuclei staining brown are positive for Ki-67. **h.** Pooled data for the semiquantitative analysis for intensity of Ki-67 staining in the vessel wall of the outflow vein specimens removed from *Iex-1* KO and WT mice. There is a significant decrease in the mean Ki-67 staining in the *Iex-1* KO animals when compared to WT controls (P<0.05). **i.** Representative staining for TUNEL from the outflow vein removed from *Iex-1* KO (**second column**) and WT mice (**third column**). Negative control is shown where the recombinant terminal deoxynucleotidyl transferase enzyme was omitted in **first column**. Nuclei staining brown are positive for TUNEL. **j.** Pooled data from the semiquantitative analysis for intensity of TUNEL staining in the vessel wall of the outflow vein specimens removed from *Iex-1* KO and WT mice. There is a significant decrease in the mean TUNEL staining in the *Iex-1* KO animals when compared to WT controls (P<0.05). Two-way Student *t*-test with post hoc Bonferroni's correction was performed. Significant difference from control value is indicated by * P<0.05. Each bar shows mean ± SEM of 4–5 animals per group. For all representative sections * denotes vessel lumen. 40X magnification, scale bar is 100-µms.

Fibroblast specific protein-1 (Fsp-1), a marker for fibroblasts, was used to determine if the fibroblast density was decreased in *Iex-1* ^−/−^ mice when compared to WT controls (**Fig. 4a**). Brown staining cells are positive for Fsp-1. The average Fsp-1 staining was significantly lower in the venous stenoses removed from *Iex-1* ^−/−^ mice when compared to WT controls (average decrease: 31%, 8.85±2.41 vs. 12.8±4.1, *Iex-1* ^−/−^ vs. WT, respectively, P<0.05, **Fig. 4b**). Myofibroblasts, α-smooth muscle actin positive cells [α-SMA (+)] are implicated in AVF failure [15], [25], [32], [33]. We assessed the number of such cells (brown staining cells, **Fig. 4c**) present in AVF venous stenoses using immunostaining. There was a significant decrease in the average α-SMA staining cells present in venous stenoses removed from *Iex-1* ^−/−^ mice when compared to WT controls (average decrease: 31%, 23.2±1.2 vs. 28.7±2.5, *Iex-1* ^−/−^ vs. WT, respectively, P<0.05, **Fig. 4d**). This change was localized primarily to the neointima. We next assessed smooth muscle heavy chain or smoothelin staining and observed no difference between the two groups (data not shown). Finally, we assessed for presence of monocytes using Ly6C immunostaining (**Fig. 4e**). There was a significant decrease in the average Ly6C staining cells present in venous stenoses removed from *Iex-1* ^−/−^ mice when compared to WT controls (average reduction: 48%, 20±6.9 vs. 38.4±8.4, *Iex-1* ^−/−^ vs. WT, respectively, P<0.05, **Fig. 4f**).

### *Iex-1* knockout mice have decreased proliferation and increased apoptosis at 28 days after AVF placement

Cellular proliferation is increased in venous stenosis formation and *Iex-1* is involved in proliferation. This was assessed using Ki-67 (nuclei staining brown) staining 28 days after AVF placement [15], [23], [25]. This revealed that the average Ki-67 index (**Fig. 4g**) among the *Iex-1* ^−/−^ group was significantly lower than the WT controls (average decrease: 69%, 1.6±0.14 vs. 6.8±0.3, *Iex-1* ^−/−^ vs. WT, respectively, P<0.001, **Fig. 4h**). Next, we used TUNEL (TdT-mediated dNTP nick end labeling) staining since *Iex-1* is involved in apoptosis [34]–[37]. This revealed a significant increase in the number of TUNEL positive cells (nuclei staining brown, **Fig. 4i**) among the venous stenosis removed from *Iex-1* ^−/−^ mice when compared to WT controls (average increase: 157%, 21.4±2.6 vs. 13.6±1.8, P<0.05, respectively, **Fig. 4j**). Overall, these results indicate that venous stenosis removed from *Iex-1* ^−/−^ mice has decreased proliferation and increased apoptosis when compared to WT controls.

### *Iex-1* and *Vegf-A* gene expression is increased in hypoxic fibroblasts

**Figure 5:**
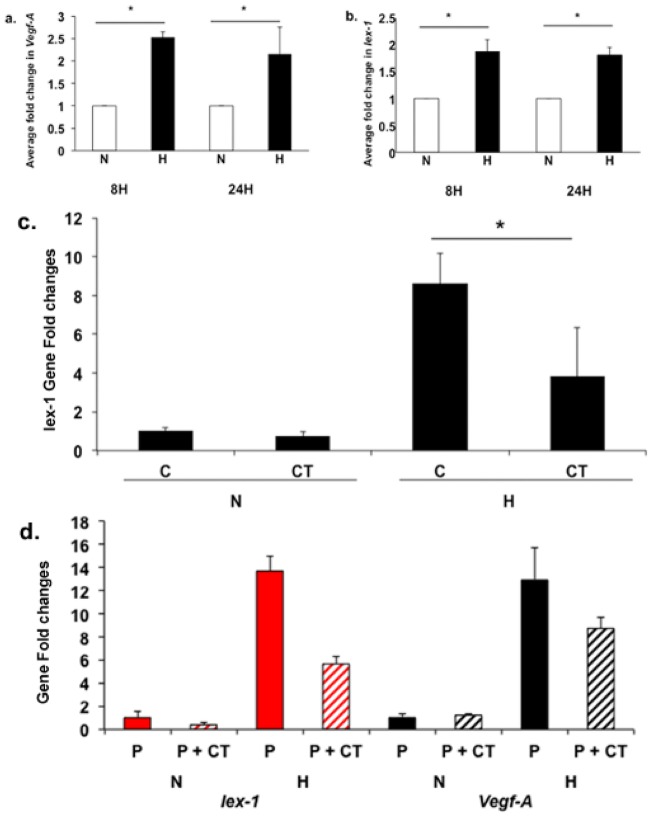
*Vegf-A* and *Iex-1* expression in hypoxic NIH 3T3 cells. Pooled data for *Vegf-A* (**a**) and *Iex-1* (**b**) expression by qRT-PCR in NIH 3T3 cells at 8 (**8H**) and 24 hours (**24H**) of normoxia (**N**) and hypoxia (**H**). **a.** Average *Vegf-A* gene expression normalized to normoxia at the same time point. **b.** Average *Iex-1* gene expression normalized to normoxia at the same time point. There is a significant increase in both the average *Vegf-A* and *Iex-1* expression in hypoxic fibroblasts when compared to normoxic (both time points, P<0.05). The average *Vegf-A* expression is increased in hypoxia when compared to normoxia at 8 and 24 hours (both time points, P<0.05). **c.** *Iex-1* expression by qRT-PCR in NIH 3T3 cells under normoxia (**N**) and hypoxia (**H**) at 24 hours with PBS alone (**C**) and calcitriol (**CT**, 1-µM). There is a decrease in the average *Iex-1* expression in **C** vs. **CT** in the hypoxia group (P<0.05). Pooled data for *Iex-1* and *Vegf-A* expression by qRT-PCR in NIH 3T3 cells at 24 hours of control (**C**, nano PLGA alone) or Calcitriol (**P+CT**, 10-µM loaded onto nano PLGA) of normoxia (**N**) and hypoxia (**H**) is shown in **d**. There is a significant decrease in both *Iex-1* and *Vegf-A* gene expression in fibroblasts treated with nano PLGA particles coated with calcitriol when compared to controls (P<0.05). Each bar shows the mean ± SEM of 3 samples per group. Two-way Student *t*-test with post hoc Bonferroni's correction was performed. Significant difference from control value is indicated by * P<0.05.

Previously, we have used a hypoxic fibroblast model to understand the mechanisms contributing to VNH formation [11], [15], [25]. Using this model, we determined the expression of *Iex-1* and *Vegf-A* at 8 and 24 hours of hypoxia and normoxia. This demonstrated that there was a significant increase in both *Iex-1* and *Vegf-A* gene expression at 8 and 24 hours of hypoxia when compared to normoxia (*Vegf-A*: 8 h, average increase: 253%, 2.53±0.1 vs. 1.0±0.00, hypoxia vs. normoxia, respectively, P<0.05, 24 h: average increase: 220%, 2.2±0.6 vs. 1.0±0.00, hypoxia vs. normoxia, respectively, P<0.05, **Fig. 5a**, *Iex-1*: 8 h, average increase: 150%, 1.5±0.2 vs. 1.0±0.02, hypoxia vs. normoxia, respectively, P<0.05, 24 h: average increase: 230%, 2.3±0.2 vs. 1.0±0.02, hypoxia vs. normoxia, respectively, P<0.05, **Fig. 5b**).

### Calcitriol reduces *Iex-1* and *Vegf-A* expression in hypoxic NIH 3T3

Calcitriol has been shown to decrease gene expression of *Iex-1* [38]–[40]. The optimal dose of calcitriol required to reduce *Iex-1* expression, 1-µM, was determined using a dose response curve. Using 1-µM of calcitriol, we determined the gene expression of *Iex-1* in hypoxic fibroblasts compared to controls at 24 hours. This demonstrated that there was a significant decrease in the average *Iex-1* expression in calcitriol treated cells when compared to controls (average decrease: 56%, 3.84±2.2 vs. 8.62±1.52, P<0.05, **Fig. 5c**). Next, we loaded calcitriol in nanoparticles composed of PLGA and determined the elution characteristics over time which demonstrated that calcitriol was eluting for up to 4-weeks (**Fig. S1**). We determined the use of these nanoparticle-loaded calcitriol compared to controls in hypoxic NIH 3T3 and determined the expression of *Iex-1* and *Vegf-A*. This demonstrated a significant reduction in both the mean *Iex-1* and *Vegf-A* gene expression in hypoxic fibroblasts treated with nano PLGA coated calcitriol compared with controls (*Iex-1*: average decrease: 58%, 5.6±0.64 vs. 13.7±1.3, P<0.05, *Vegf-A*: average decrease: 33%, 8.74±0.92 vs. 12.9±2.81, P<0.05, **Fig. 5d**).

### Adventitial delivery of calcitriol to the outflow vein of AVF reduces *Iex-1*, *Vegf-A*, and *Mcp-1* expression 7 days later with significant reduction in the neointima area at day 28

**Figure 6:**
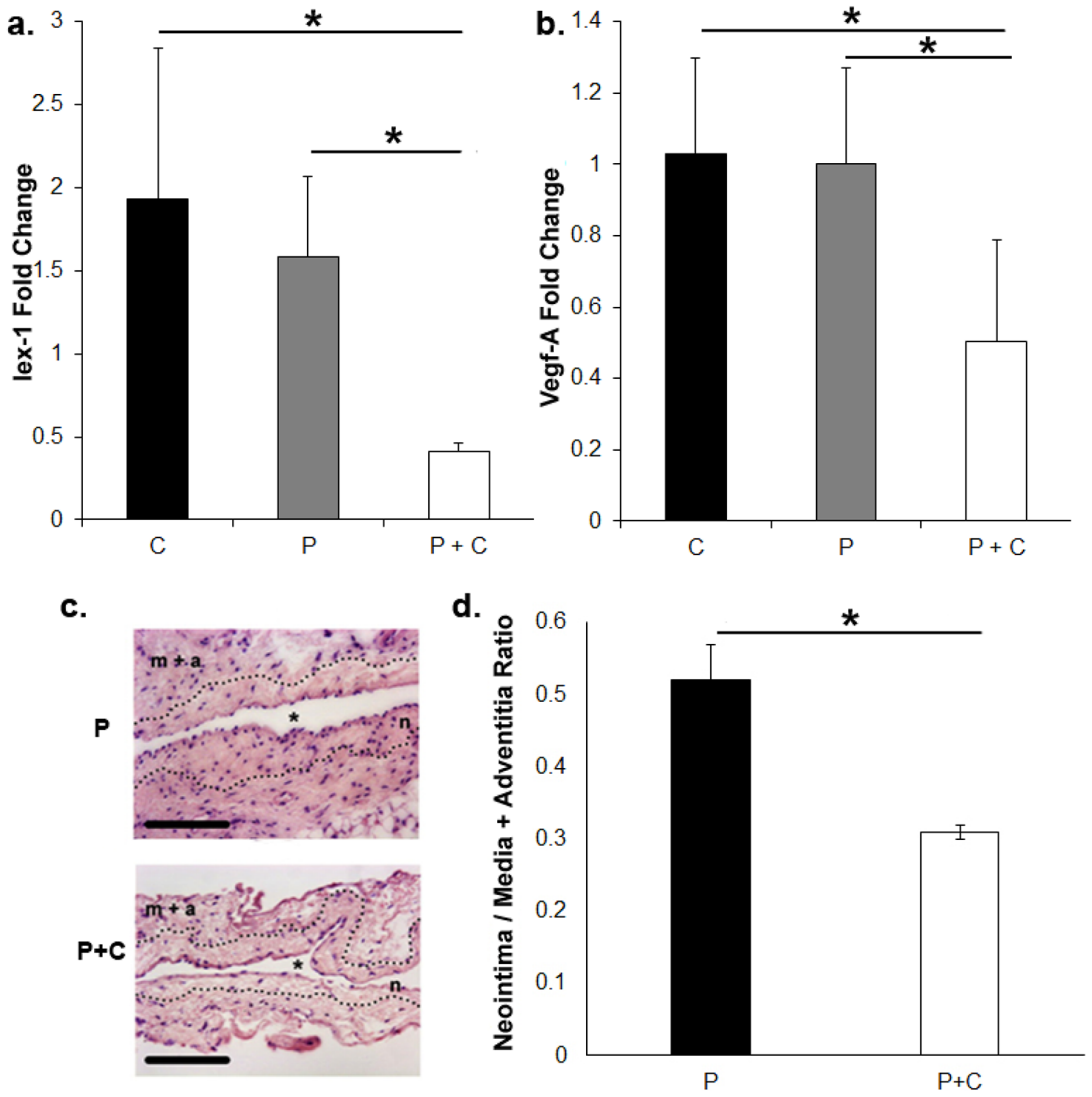
Gene expression of *Iex-1* and *Vegf-A* by qRT-PCR in outflow vein of AVF seven days after adventitial delivery of hydrogel alone, hydrogel with nanoparticle PLGA without calcitriol, or hydrogel with calcitriol in nanoparticle PLGA. **a.** Pooled data for *Iex-1* expression by qRT-PCR in outflow veins of AVF seven days after adventitial delivery of hydrogel alone (**C**), hydrogel with nanoparticle PLGA without calcitriol (**P**), or hydrogel with calcitriol in nanoparticle PLGA (100-µM, **P+C**). This demonstrates a significant decrease in the mean *Iex-1* expression in nanoparticle coated with calcitriol compared to PLGA (P<0.05). **b.** Pooled data for *Vegf-A* expression by qRT-PCR in outflow veins treated of AVF seven days after adventitial delivery of hydrogel alone (**C**), hydrogel with nanoparticle PLGA without calcitriol (**P**), or hydrogel with calcitriol in nanoparticle PLGA (100-µM, **P+C**). This demonstrates a significant decrease in the mean *Vegf-A* gene expression in nanoparticle coated with calcitriol compared to PLGA alone (P<0.05). **c.** Representative hematoxylin and eosin staining of the outflow vein removed from PLGA (**P**) and PLGA + Calcitriol (**P+C**) from 28 days after fistula placement. The neointima (**n**) is identified from the media and adventitia by the dotted line. **m+a** is the media and adventitia. * indicates lumen. 40X magnification, scale bar is 100-µms. **d.** Neointima area/Media + adventitia area ratios for the **P** and **P+C** group. There was a significant reduction in the average neointima to media plus adventitia ratio in the calcitriol treated vessels when compared to PLGA alone (P<0.05). Two-way Student *t*-test with post hoc Bonferroni's correction was performed. Significant difference from control value is indicated by * P<0.05. Each bar shows mean ± SEM of 4-6 animals per group.

Adventitial delivery of nanoparticle composed of PLGA coated with calcitriol to the outflow vein of AVF placed in mice with established CKD was performed to assess the expression of *Iex-1*. At 7 days after AVF placement, there was a significant reduction in the mean *Iex-1* gene expression in nanoparticle PLGA coated calcitriol when compared to controls (average decrease: 35%, 0.42±0.01 vs. 1.7±0.01 vs. 2.67±0.01, PLGA + Calcitriol, PLGA, and PBS, respectively, P<0.05, **Fig. 6a**). In addition, we observed a significant decrease in the *Vegf-A* expression as well (average decrease: 35%, 0.42±0.01 vs. 1.7±0.01 vs. 2.67±0.01, PLGA + Calcitriol, PLGA, and PBS, respectively, P<0.05, **Fig. 6b**). Overall, these results indicate that calcitriol can be used to decrease *Iex-1* expression in both *in vitro* and *in vivo* and therefore used as a translational therapy for reducing *IEX-1* expression.

We assessed the changes in the venous outflow vein using histomorphometric analysis at day 28. This demonstrated that there was a significant reduction in the average neointima to media plus adventitia ratio in the calcitriol treated vessels when compared to PLGA alone (average decrease: 34%, PLGA + Calcitriol vs. PLGA, respectively, P<0.05, **Figs. 6c and d**).

## Discussion

Currently, more than 570,000 patients have ESRD in the United States [1]. The majority of the patients requires hemodialysis and need a well-functioning vascular access to allow for adequate purification of their blood [1]. Arteriovenous fistula, which is the preferred vascular access, has a one-year patency of 60% and the majority of fistulas fail because of venous stenosis formation as a result of VNH. In the current study, we describe a new gene, *Iex-1*, which was found to be increased in venous outflow segments removed from patients with AVF. We demonstrate in venous stenosis removed from *Iex-1* ^−/−^ mice when compared to WT animals: 1). Increase in lumen vessel area with a decrease in neointima area and accompanying cell density; 2). a decrease in fibroblast, α-SMA, and Ly6C expression; 3). A decrease in cellular proliferation with increase in apoptosis; and a 4) reduction in expression of *Vegf-A*, *Mcp-1*, and *MMP-9* expression. Finally, we demonstrate nanoparticles composed of PLGA loaded with calcitriol can reduce *Iex-1* expression *in vivo* and *in vitro* and therefore used as a translational therapy.

There are multiple factors, which are felt to contribute to venous neointimal hyperplasia. These include inflammatory oxidative stress caused by the dialysis environment [8]. Studies have demonstrated that high oxidative stress associated with dialysis is responsible for elaboration of growth factors and cytokines. Others hypothesis includes changes in blood flow, shear stress, and elastic mismatch [6], [41], [42]. Recently, a prospective study in patients with hemodialysis vascular access demonstrated that increased red blood cell distribution width was associated with long-term hemodialysis AVF failure at 2 years [43].

*Iex-1* is a cytoplasmic and nuclear glycosylated protein that has an important role in controlling cellular growth and apoptosis [19], [20], [38]. *Iex-1* is induced by X-irradiation, ultraviolet B (UVB)-irradiation, growth factors and chemical reagents such as epidermal growth factor, pituitary adenylate cyclase activating peptide (PACAP), tumor promoting phorbol ester (TPA), and intra-cellular mediators such as Sp1 and NFκβ [21], [36], [38], [44]–[47]. Work by Shen et al has shown IEX-1 targets the mitochondrial F1Fo-ATPase Inhibitor (IF1) for degradation when the cell is stressed [48]. This results in increased ATP hydrolysis and a reduction in ROS. However, this understanding does not account for the differential effects of IEX-1 in a variety of cell types and stimuli. The gene is repressed by factors that are known to inhibit cellular growth such as transforming growth factor-β (TGF-β), calcitriol (1,25-dihydroxyvitamin D_3_) and intra-cellular growth inhibitory molecules such as p53 [19], [20], [38], [46]. A deletion analysis of the *Iex-1* promoter has identified 1,25-dihydroxyvitamin D_3_ response elements within the promoter, in addition to other elements including Sp1 and p53 response elements.

The mouse ortholog of the *Iex-1* gene is known as *Iex-1*/gly96. The knock out mouse was generated using an *Iex-1*/gly96 null mutant mouse [27]. AVFs were placed in *Iex-1* ^−/−^ and WT mice with pre-existing CKD and animals were sacrificed at 28 days after AVF placement for histomorphometric analysis. In *Iex-1* ^−/−^ mice when compared to WT, there was an increase in the average lumen vessel area with a decrease in neointima area. In addition, there was a decrease in fibroblasts, myofibroblasts, and Ly6C positive cells accompanied with a decrease in proliferation, and an increase in TUNEL. The findings of increase in TUNEL staining and decrease in cellular proliferation are consistent with other studies that have shown that *Iex-1* alters the rate of cell growth and apoptosis in various cells [23], [34], [36], [37]. Cellular over-expression of *Iex-1* is associated with an increase in the growth rate of normal keratinocytes and HeLa cells [19], [36], [49]. In keratinocytes and vascular smooth cells, an increase in the rate of apoptosis has been observed and associated with changes in *Iex-1* [23], [34], [37], [49].

The finding of positive vascular remodeling in the *Iex-1* ^−/−^ mice when compared to WT animal is new. There are limited studies studying the role of *Iex-1* in vascular injury. A previous study demonstrated that over expression of *Iex-1* to the endothelium using an adenoviral vector in a murine carotid artery injury model using an apo-E knockout mouse results in decreased intima to media ratio [23]. In this study, the authors postulated that the effect was mediated through monocytes. In addition, they demonstrated that in atherosclerotic plaque specimens removed from patients, there was increased *Iex-1* expression [23]. In the present study, we demonstrated a decrease in Ly6C (+) monocytes. Ly6C (+) cells have been associated with vascular injury caused by atherosclerosis [50]. The expression of *Iex-1* in AVF was not known and we observed a significant increase in the mean *Iex-1* staining in the adventitial region when compared to control of venous segments removed from patients with AVF.

Previous study from our laboratory demonstrated that reducing *Vegf-A* gene expression in mice with AVF results in a decrease in neointima with positive vascular remodeling, a decrease in proliferation, and an increase in TUNEL [15], [25]. Therefore, we hypothesized that in venous stenosis removed from *Iex-1* ^−/−^ mice when compared to WT animals; there would be a decrease in *Vegf-A* expression. The expression of *Vegf-A* was first assessed using *Vegf-A* staining which demonstrated a significant decrease in *Vegf-A* expression at 28 days after AVF placement [15], [25]. Moreover, we determined the gene expression of *Vegf-A* using qRT-PCR analysis and observed a significant decrease in *Vegf-A* expression in *Iex-1* ^−/−^ mice when compared to WT animals. We also observed a decrease in the gene expression of *Mcp-1* using qRT-PCR analysis and observed a significant decrease in *Mcp-1* expression as well. Previous studies have demonstrated that reducing *Mcp-1* expression is associated with a reduction in VNH [16]. Additionally, we also observed a decrease in MMP-9 expression consistent with previous work from our laboratory [15]. These data suggest that *Iex-1* ^−/−^ mice have decreased expression of *Vegf-A*/*Mcp-1* axis accompanied with a subsequent downstream reduction in MMP-9.

Movement of the *Iex-1* protein in and out of the nucleus is controlled by a variety of factors such as 1α,25 (OH)_2_ D_3_ and other biologic and physical factors [19], [21], [23]. We have previously used the hypoxic fibroblast to myofibroblast *in vitro* assay to investigate the mechanisms of VNH associated with AVF failure [11]. We wanted to develop a translatable therapy for reducing *Iex-1* expression and we used calcitriol, which reduces *Iex-1* expression. We demonstrated that using nanoparticle coated with calcitriol reduces hypoxia induced up regulation of both *Iex-1* and *Vegf-A*. Nanoparticles composed of PLGA have been used for local drug delivery. We wanted to target the adventitia of the outflow vein at the time of fistula placement because there was a reduction in the Fsp-1 staining in the Iex-1 KO animals. Our laboratory and others have performed adventitial delivery to the outflow vein in the past to reduce VNH associated with AVF [15], [29].

We performed *in vitro* experiments, which demonstrated the optimal dose for reducing *Iex-1* expression to be 1-µM, which is consistent with other studies [18], [19]. Based on this data, we loaded a 200 times more calcitriol into PLGA particles at a concentration of 200-µM and found the calcitriol eluted for 4-weeks. Hydrogel loaded nanoparticles coated with calcitriol delivered to the adventitia of the outflow vein of AVF in mice with established CKD demonstrated a significant reduction in both *Iex-1* and *Vegf-A* expression by qRT-PCR at 7 days later. We determined the response on vascular remodeling at day 28 by performing histomorphometric analysis and found that there was a significant reduction in the average neointima area to media and adventitia area in the calcitriol treated vessels when compared to controls. The use of nanoparticle composed PLGA is a novel therapy and has not been used for local drug delivery in vascular injury models but can be used for local delivery to the adventitia.

There are several limitations of the present study. One is we used a knockout mouse which is not cell specific and therefore the observations made in venous stenosis formation could be attributed to other cellular phenotypes such as leukocytes, neutrophils, platelets, and circulating progenitor cells. Second, the mouse model does not entirely capitulate the human disease. Finally, the observation of IEX-1 expression was made in small number of human samples and larger studies are needed to add greater power to these observations.

**Figure 7:**
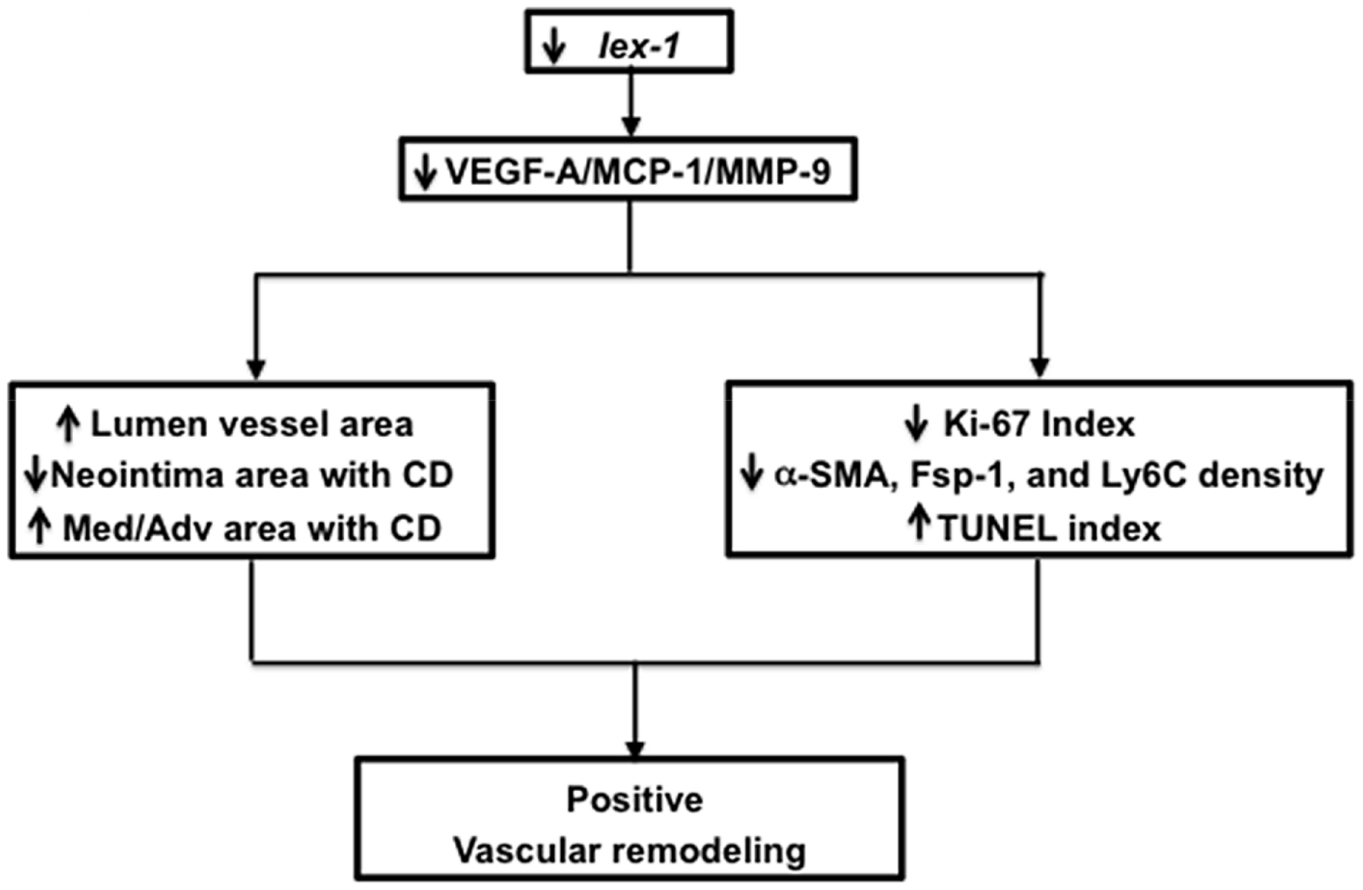
Synopsis of major findings.

In conclusion, the present study demonstrates that *Iex-1* is implicated in VNH associated with AVF. By eliminating or reducing *Iex-1* expression, we hope to extend AVF patency by reducing inflammatory and angiogenic responses to cell stress. Decreasing these responses through *Iex-1* would limit *Vegf-A*/*Mcp-1* and *Mmp-9* mediated VNH (**Fig. 7**). These changes result in a decrease in proliferation, an increase in apoptosis and increased positive vascular remodeling. These findings suggest that *Iex-1* is a viable target for the development of translational therapies designed to inhibit VNH in patients with AVF among the ESRD population.

## Supporting Information

Figure S1 Calcitriol elution curve from nanoparticle coated PLGA over time.
(PPT)
