# Correction: The Role of *Iex-1* in the Pathogenesis of Venous Neointimal Hyperplasia Associated with Hemodialysis Arteriovenous Fistula

**DOI:** 10.1371/journal.pone.0110196

**Published:** 2014-09-30

**Authors:** The PLOS ONE Staff

There are errors in one of the co-author’s name and the funding statement. The sixth author's name is spelled incorrectly. The correct name is: Min Kyung Lee.

Additionally, there is a missing grant number in the funding statement. Please refer to the correct funding statement below:

This work was funded by a R01HL098967 (SM) and 1R01 HL109192 from the National Heart, Lung, And Blood Institute, Society of Interventional Radiology Foundation (AB), and Howard Hughes Medical Institute (AB). The funders had no role in study design, data collection and analysis, decision to publish, or preparation of the manuscript.
