# Supplementary figures and images for: The Role of Iex-1 in the Pathogenesis of Venous Neointimal Hyperplasia Associated with Hemodialysis Arteriovenous Fistula
Source: PLoS One. 2014 Jul 18;9(7):e102542. doi: 10.1371/journal.pone.0102542 (PMC4103828; doi:10.1371/journal.pone.0102542)

## Slide 1
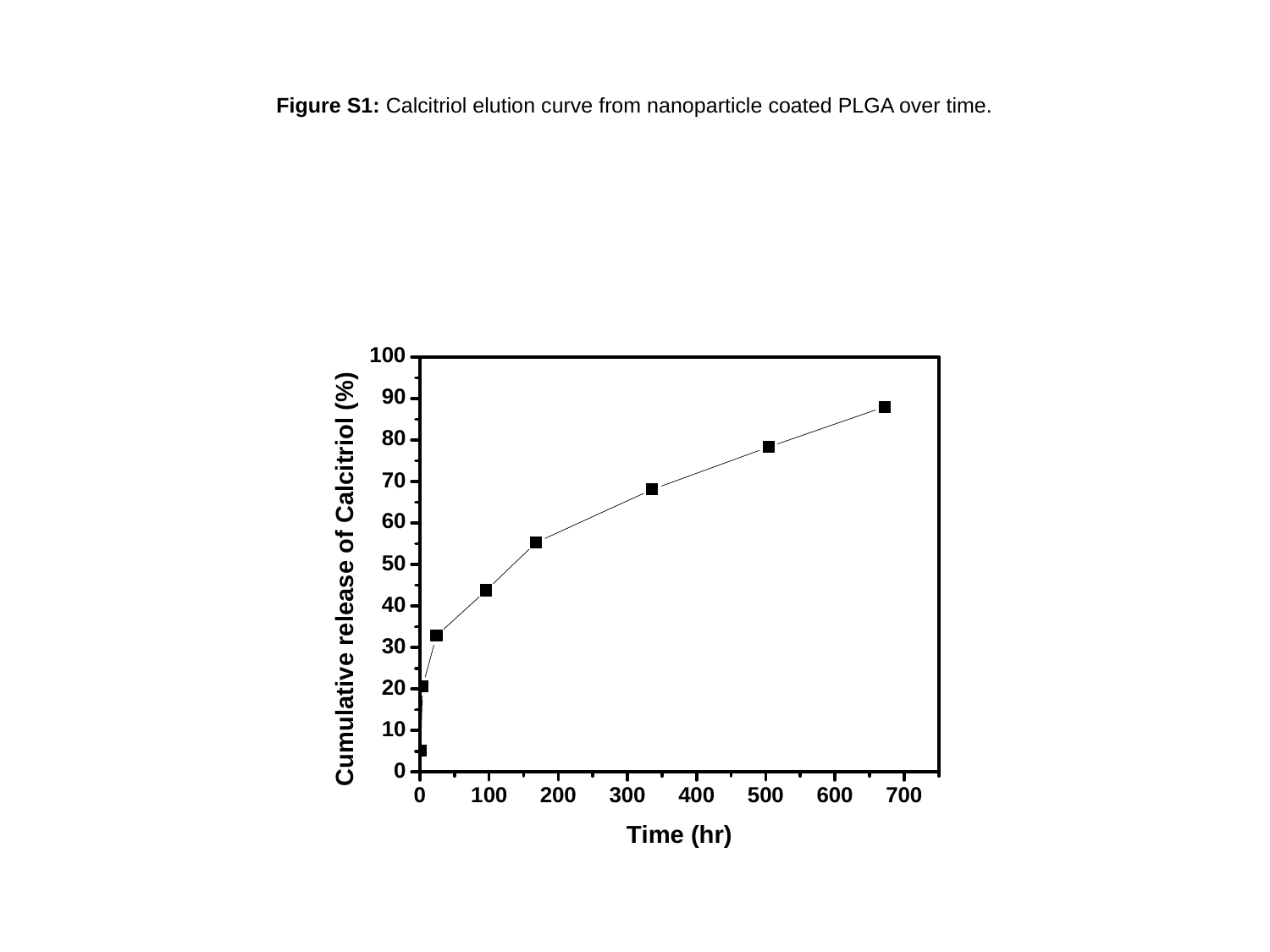

# Figure S1: Calcitriol elution curve from nanoparticle coated PLGA over time.

Supplement: Figure S1 — Calcitriol elution curve from nanoparticle coated PLGA over time. (PPT) [file pone.0102542.s001.ppt]
